# The mode and timing of administrating nutritional treatment of critically ill elderly patients in intensive care units: a multicenter prospective study

**DOI:** 10.3389/fmed.2024.1321599

**Published:** 2024-02-07

**Authors:** Wei Chen, Milin Peng, Zhiwen Ye, Yuhang Ai, Zhiyong Liu

**Affiliations:** ^1^Department of Critical Care Medicine, Xiangya Hospital, Central South University, Changsha, Hunan, China; ^2^National Clinical Research Center for Geriatric Disorders, Xiangya Hospital, Central South University, Changsha, Hunan, China; ^3^Hunan Provincial Clinical Research Center for Critical Care Medicine, Xiangya Hospital, Central South University, Changsha, Hunan, China

**Keywords:** enteral nutrition, parenteral nutrition, elderly, intensive care units, mortality

## Abstract

**Introduction:**

Critically ill patients are more susceptible to malnutrition due to their severe illness. Moreover, elderly patients who are critically ill lack specific nutrition recommendations, with nutritional care in the intensive care units (ICUs) deplorable for the elderly. This study aims to investigate nutrition treatment and its correlation to mortality in elderly patients who are critically ill in intensive care units.

**Method:**

A multiple-center prospective cohort study was conducted in China from 128 intensive care units (ICUs). A total of 1,238 elderly patients were included in the study from 26 April 2017. We analyzed the nutrition characteristics of elderly patients who are critically ill, including initiated timing, route, ways of enteral nutrition (EN), and feeding complications, including the adverse aspects of feeding, acute gastrointestinal injury (AGI), and feeding interruption. Multivariate logistic regression analysis was used to screen out the impact of nutrition treatment on a 28-day survival prognosis of elderly patients in the ICU.

**Result:**

A total of 1,238 patients with a median age of 76 (IQR 70–83) were enrolled in the study. The Sequential Organ Failure (SOFA) median score was 7 (interquartile range: IQR 5–10) and the median Acute Physiology and Chronic Health Evaluation (APACHE) II was 21 (IQR 16–25). The all-cause mortality score was 11.6%. The percentage of nutritional treatment initiated 24 h after ICU admission was 58%, with an EN of 34.2% and a parenteral nutrition (PN) of 16.0% in elderly patients who are critically ill. Patients who had gastrointestinal dysfunction with AGI stage from 2 to 4 were 25.2%. Compared to the survivors’ group, the non-survivors group had a lower ratio of EN delivery (57% vs. 71%; *p* = 0.015), a higher ratio of post-pyloric feeding (9% vs. 2%; *p* = 0.027), and higher frequency of feeding interrupt (24% vs. 17%, *p* = 0.048). Multivariable logistics regression analysis showed that patients above 76 years old with OR (odds ratio) 2.576 (95% CI, 1.127–5.889), respiratory rate > 22 beats/min, and ICU admission for 24 h were independent risk predictors of the 28-day mortality study in elderly patients who are critically ill. Similarly, other independent risk predictors of the 28-day mortality study were those with an OR of 2.385 (95%CI, 1.101–5.168), lactate >1.5 mmol/L, and ICU admission for 24 h, those with an OR of 7.004 (95%CI, 2.395–20.717) and early PN delivery within 24 h of ICU admission, and finally those with an OR of 5.401 (95%CI, 1.175–24.821) with EN delivery as reference.

**Conclusion:**

This multi-center prospective study describes clinical characteristics, the mode and timing of nutrition treatment, frequency of AGI, and adverse effects of nutrition in elderly ICU patients. According to this survey, ICU patients with early PN delivery, older age, faster respiratory rate, and higher lactate level may experience poor prognosis.

## 1 Introduction

With the global increase in elderly patients who are critically ill, hospital admissions also increased, especially at the peak of the COVID-19 infection (1). With increasing aging, symptoms of critical illness in elderly people become more significant, alongside poor clinical outcomes attributed to several intensive care unit (ICU) factors (2). With the onset of organ degradation and poor immune function in elderly patients, malnutrition contributes significantly to poor clinical outcomes, including increased incidence of infections, length of hospital and ICU stay, and risk of mortality, among others (3).

Malnutrition is defined as the state of insufficient intake or uptake of nutrients, leading to an altered body composition (2). The study by Agarwal et al. evaluated 3,122 patients with a mean age of 64.6 ± 18 years and concluded that participants suffering from malnutrition were 41% (4). In critically ill elderly patients, malnutrition was higher with poor outcomes, increased rates of infections, length of hospital stay, and mortality risks (5). More data are required to evaluate nutrition in critically ill elderly patients.

Most critically ill patients, especially the elderly, require artificial nutrition. It is reported that neither higher nor lower energy intake improves clinical outcomes in critically ill elderly patients (6). However, food intake between 12 and 25 kcal/kg in the first 7–10 days of ICU stay is recommended (7). However, a decrease in fat-free body mass and resting energy expenditure (REE) generally decreases due to aging. Approximately 30 kcal/kg body weight is a recommended rough estimate and general orientation for energy requirements in older persons (8). In addition, for nutrition support therapy, early enteral nutrition (EN) is beneficial for maintaining gut integrity, modulating stress, and regulating the systemic immune system. Due to higher rates of gastrointestinal (GI) dysfunction in elderly critical patients, clinical nutrition therapy has changed substantially.

Owing to poor nutrition recommendations and malnutrition in critically ill elderly patients and the consequent longer hospital stays, poor clinical outcomes, and high mortality risks (9, 10), it is important to develop protocols to improve nutrition treatments in elderly critical patients. This study aims to evaluate the clinical characteristics of nutrition therapy and the impact of nutrition on mortality in elderly critical patients.

## 2 Materials and methods

### 2.1 Methods

#### 2.1.1 Study design

A multiple-center prospective observational cohort study was conducted in China from 128 intensive care units (ICUs) in 116 hospitals. The clinical information of patients was collected on the first day of ICU admission, and the nutritional tolerance of the patients was assessed to choose the appropriate nutritional therapy. A follow-up visit was made to assess the survival prognosis of the patients on the 28th day of ICU admission. The aim was to explore the effect of nutrition treatment on 28-day survival outcomes in elderly patients during ICU admission. Patients who were admitted to ICU on 26 May 2017 were screened for eligibility based on the following inclusion criteria: (A) first-time ICU admission patients; (B) above 65 years old and more than 7 days of stay in the ICU; and (C) patients with approximately 2 months without gastrointestinal surgery. The exclusion criteria were as follows: (A) patients with severe cachexia; (B) severe craniocerebral injury patients with lack of consciousness; (C) patients without informed consent; and (D) patients without follow-up within 28 days after ICU admission. The flowchart is shown as a Supplementary Figure S1. A total of 1,238 patients were included in our study. Survival outcomes at day 28 were used as the endpoint, and clinical characteristics and variables were collected and recorded. All data involved in our study were obtained from a customized website and authorized by the ethics committee of Nanjing Hospital (no. 2017NZKY-010-01).

### 2.2 Data collection

#### 2.2.1 Baseline characteristics

Clinical information was collected on the first day during ICU admission, including age, sex, weight, height, comorbidities, and diagnoses. Vital signs were also collected, including body temperature, mean arterial pressure, heart rate, and respiratory rate. Routine laboratory tests were carried out, including white blood cell (WBC) count, percentage of lymphocytes, platelet, total bilirubin, albumin, creatinine, C-reactive protein (CRP), maximum and minimum blood glucose, oxygenation, and lactate. Acute Physiology and Chronic Health Evaluation II (APACHE II) score and Sequential Organ Failure Assessment (SOFA) score were assessed. All these baseline characteristics were collected on the first day of ICU admission. All clinical data were collected with the informed consent of patients and their families (including the study protocol, study purpose, and study content), and all clinical data were identified to protect patients’ privacy.

##### 2.2.1.1 Nutrition-associated parameters

We also extracted some nutrition-related indicators, such as serum albumin levels on the first day of ICU admission. Furthermore, we continuously recorded more specific information related to nutrition treatment during ICU admission, including the initiation of nutritional therapy, the mode of nutritional therapy (enteral, parenteral, or oral), and the option of enteral nutrition delivery (such as gastric feeding, post-pyloric feeding, percutaneous, or jejunostomy). At the same time, we also conducted detailed assessments of patients’ tolerance to enteral nutrition during hospitalization, including nausea, vomiting, aspiration, abdominal pain, abdominal distension, and diarrhea.

##### 2.2.1.2 AGI scale and assessment

Early enteral nutrition in critically ill patients is important for improved rehabilitation and prevention of related complications. According to the ESPEN guidelines of the intensive care unit, critically ill patients admitted to the ICU for more than 48 h are at risk of malnutrition. It was further noted that early enteral nutrition can improve gastrointestinal mucosa and prevent intestinal microbiota translocation, which should be implemented within 3–7 days (11). However, acute gastrointestinal injury (AGI) was common among critically ill patients (12). AGI is mainly manifested as gastric retention or reflux, abdominal distension, diarrhea, gastrointestinal paralysis, abdominal pressure, and gastrointestinal bleeding. Therefore, we conducted a comprehensive assessment of patient tolerance to nutritional therapy on the first day during ICU admission and selected personalized nutritional plans and methods for each patient through consecutive 8 days of evaluation of the patient’s gastrointestinal function (the final evaluation, also labeled as the eighth evaluation, was performed on day 10 after admission to the ICU) based on the AGI scale originated from the ESICM Working Group on Abdominal Problems (12).

### 2.3 Statistical analysis

The collected data were analyzed using SPSS software (version 26.0) and R version 4.0.3. Continuous variables were reported as a median and interquartile range with non-gaussian data distribution confirmed by the Kruskal–Wallis test. Continuous variables were compared using the non-parametric Mann–Whitney test. Categorical variables were compared using the chi-square test.

A receiver operating characteristic (ROC) curve was performed to calculate the threshold for predicting the long-term mortality of each continuous variable with significant differences after univariate logistics regression analysis. Logistic regression was used for univariate and multivariate analysis to calculate mortality predictive values, with odds ratio (OR) and a 95% confidence interval (CI). Variables in the univariate logistics regression analysis were selected using an enter elimination method. Multivariate logistic regression analysis was performed to adjust for several confounders, incorporating all risk factors with a *p-*value of 0.05. The selection of likelihood ratio (LR) test for maximum partial likelihood estimation (forward: LR) was used to choose independent prognostic factors using odds ratio (OR) and 95% confidence interval. All parameters with a *p-*value of 0.05 were statistically significant.

## 3 Results

### 3.1 Baseline characteristics of participants

In total, 1,238 critically ill elderly patients were enrolled in this study. The overall 28-day survival rate was 88.4% (*n* = 1,094), including 1,094 survivors and 144 non-survivors, of which 804 (65%) patients were male. The median age for patients was 76 years (IQR 70–83), height was 168 cm (IQR 160–173), and weight was 61 kg (IQR 55–70). The median SOFA and APACHE II scores were 7 (IQR 5–10) and 21 (IQR 16–25). The characteristics and variables of the study of the elderly population are presented in Table 1.

**Table 1:** Baseline clinical characteristics in elderly critically ill patients.

Variables	Total (*n* = 1,238)
Gender, male, *n* (%)	804 (65)
Age, Median (IQR), years	76 (70, 83)
Height, Median (IQR), cm	168 (160, 173)
Weight, Median (IQR), kg	61 (55, 70)
ICU diagnosis, *n* (%)	
Sepsis	104 (8)
Sepsis shock	161 (13)
Cardiac arrest	51 (4)
Severe pancreatitis	46 (4)
Cerebral disease	320 (26)
Underlying diseases, *n* (%)	
Hypertension	338 (27)
Diabetes	188 (15)
Chronic kidney dysfunction	189 (15)
Gastrointestinal tumor	116 (9)
Clinical examination during 24 h ICU admission, Median (IQR)	
Oxygenation index	200 (130, 286.5)
Platelet, × 10^9^/L	170 (120, 237)
Total bilirubin, mmol/L	13.9 (8.93, 21.87)
Creatinine, umol/L	89.65 (64.35, 134)
SOFA	7 (5, 10)
APACHE II	21 (16, 25)
Temperature, °C	37 (36.6, 37.98)
Mean arterial pressure, mmHg	80 (70, 96)
Heart rate, beats/min	100 (84.25, 119)
Respiratory rate, beats/min	22 (18, 26)
GCS	9 (6, 14)
WBC, × 10^9^/L	11.70 (8.3, 16)
lymphocytes%	7.15 (4, 12.38)
CRP, mmol/L	58.34 (19, 120)
ALB, mg/dl	30.8 (26.9, 35)
Lactate, mmol/L	1.9 (1.2, 3.1)
Glucose_min, mmol/L	6.8 (5.6, 8.5)
Glucose_max, mmol/L	11.3 (8.9, 14.8)
Day28_ICU stay, *n* (%)	358 (29)
28_day mortality, *n* (%)	144 (11.6)

IQR, interquartile range; ICU, Intensive Care Unit; SOFA, Sequential Organ Failure Assessment; APACHE II, Acute Physiology and Chronic Health Evaluation II; GCS, Glasgow coma scale; WBC, white blood cell; CRP, C-reactive protein; ALB, albumin.

### 3.2 Features of nutrition treatment in critically ill elderly patients

**Figure 1:**
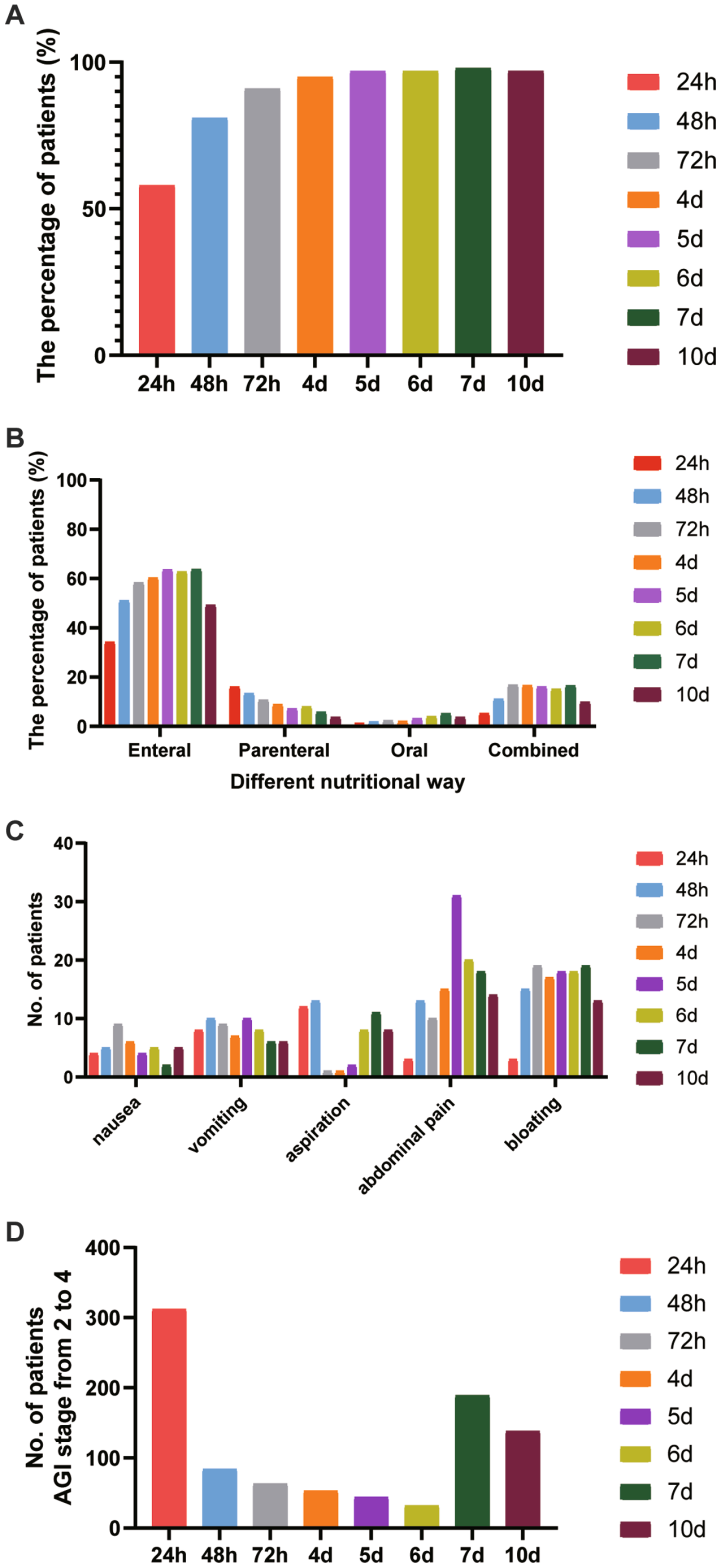
The features of nutrition treatment in elderly critically ill patients. **(A)** The percentage of nutrition treatment-initiated timing at different feeding stages from 24 h to day 10 after ICU entry. **(B)** The proportion of nutritional routines at different feeding stages from 24 h to day10 after ICU entry. **(C)** The adverse aspects of feeding treatment at different feeding stages. **(D)** The distribution of the AGI stage with grades of 2 to 4 was shown.

As shown in Figure 1A, nutrition treatment started within 24 h, 48 h, 72 h, and day 7 after ICU admission were 58% (*n* = 722), 81% (*n* = 999), 91% (*n* = 1,130), and 98% (*n* = 1,208), respectively, in elderly critically ill patients. The ratio of subjects receiving EN within 24 h after ICU admission was 34.2% (*n* = 488). This increased to 51.0% after 48 h and 63.7% after day 7. The percentage of patients receiving PN was 16.0%, 24 h after ICU admission. This decreased to 13.4% after 48 h and 5.8% after day 7. In addition, the percentage of oral treatment 24 h after ICU admission was 1.3%, and EN combined with PN was 5.3% (Figure 1B). The distribution of nutrition treatment is demonstrated in Figure 1B. The mode of EN delivery used was gastric feeding in 85% of patients, post-pyloric feeding in 2% of patients, and PEG/J or jejunostomy feeding in 2% of patients. The infusion style of EN delivery was a continuous pump in 95% of patients and an intermittent pump in 5% of patients.

Figure 1C shows the adverse effects of nutrition treatment, including nausea, vomiting, aspiration, abdominal pain, and bloating at different feeding stages. We found that at an early stage, the frequency of aspiration occurred in 12 elderly patients 24 h after ICU admission and 13 patients after 48 h. The frequency of abdominal pain occurred in 3 patients 24 h after ICU admission, increased to 13 patients after 48 h, and 18 patients on day 7.

In this study, we assessed the AGI grade in elderly critically ill patients from 24 h to day 10 after admission to ICU. The distribution of the AGI stage from 2 to 4 is shown in Figure 1D. After 24 h of ICU admission, 25.2% (*n* = 312) of elderly patients had gastrointestinal dysfunction with AGI stage from 2 to 4. It decreased to 6.8% (*n* = 84) after 48 h and increased to 15.3% (*n* = 189) on day 7 after ICU admission.

### 3.3 Features of nutrition treatment between survivors and non-survivors groups in critically elderly ill patients

We categorized the elderly patients into two groups according to the mortality 28 days after ICU admission (Table 2). Compared to the survivors’ group, the non-survivor group had significant elderly patients (78 years vs. 75 years old; *p* = 0.038), a lower rate of cerebral disease (15% vs. 27%; *p* = 0.003), and a higher rate of gastrointestinal tumor (19% vs. 7%; *p* = 0.001). We also assessed the clinical characteristics 24 h after ICU admission in these two groups, and the results showed that, compared to the survivors’ group, the non-survivors group had higher SOFA scores (8.5 vs. 7 years old; *p* = 0.01) and APACHE II score (23 vs. 20; *p* = 0.002); higher frequencies of heart rate (109 vs. 100; *p* = 0.027) and respiratory rate (24 vs. 22; *p* = 0.004); higher levels of serum CRP (82 vs. 55 mg/dL; *p* = 0.003) and serum lactate (2.2 vs. 1.8 mmoL/L; *p* < 0.001); and lower level of serum platelet (158 vs. 172 × 10*9/L; *p* = 0.015) and the percentage of lymphocyte (6.5 vs. 7.3%; *p* = 0.033).

**Table 2:** Clinical characteristics in elderly patients are compared for survivor and non-survivor groups.

Variables	Survivor group (*n* = 1,094)	Non-survivor group (*n* = 144)	*p*
Gender, *n* (%)	704 (64)	100 (69)	0.266
Age, Median (IQR), years	75 (70, 82)	78 (70.75, 84)	0.038
Height, Median (IQR), cm	168 (160, 173)	170 (160, 173)	0.276
Weight, Median (IQR), kg	62 (55, 70)	60 (55, 68)	0.057
ICU diagnosis, *n* (%)			
Sepsis	92 (8)	12 (8)	1
Sepsis shock	141 (13)	20 (14)	0.839
Cardiac arrest	48 (4)	3 (2)	0.278
Severe pancreatitis	40 (4)	6 (4)	0.944
Cerebral disease	298 (27)	22 (15)	0.003
Underlying diseases, *n* (%)			
Hypertension	302 (28)	36 (25)	0.575
Diabetes	164 (15)	24 (17)	0.687
Chronic liver dysfunction	40 (4)	6 (4)	0.944
Chronic kidney dysfunction	167 (15)	22 (15)	1
Gastroenteric tumor	98 (7)	18 (19)	0.001
Clinical examination during 24 h ICU admission, Median (IQR)			
SOFA	7 (5, 10)	8.5 (6, 11)	0.010
APACHEII	20 (16, 25)	23 (17, 27)	0.002
Temperature, °C	37 (36.7, 37.98)	37 (36.5, 37.92)	0.492
Mean arterial pressure, mmHg	80 (70, 96)	78.5 (68, 91.25)	0.256
Heart rate, beats/min	100 (84, 118)	109 (86, 121)	0.027
Respiratory rate, beats/min	22 (18, 26)	24 (19, 28.25)	0.004
GCS	9 (6, 13)	8.5 (5, 14)	0.714
Oxygenation index	200 (132, 290)	184 (122, 265)	0.268
Platelet, × 10^9^/L	172 (121, 241)	158 (100, 207)	0.015
Total bilirubin, mmol/L	13.9 (8.9, 21.98)	14.35 (9.4, 20.65)	0.769
Creatinine, umol/L	88.5 (64.03, 132)	94.16 (67.75, 156.62)	0.160
WBC, × 10^9^/L	11.7 (8.22, 16)	12.15 (8.83, 15.8)	0.720
lymphocytes%	7.3 (4.1, 12.5)	6.5 (3.68, 10.83)	0.033
CRP, mmol/L	55 (18, 117)	82 (33, 149)	0.003
ALB, mg/dl	31 (27, 35.1)	29.56 (25.95, 33.5)	0.026
Lactate, mmol/L	1.8 (1.2, 3)	2.2 (1.6, 4.0)	<0.001
Glucose_min, mmol/L	6.8 (5.6, 8.5)	6.8 (5.68, 8.45)	0.901
Glucose_max, mmol/L	11.3 (8.9, 14.88)	11.5 (8.97, 14.2)	0.962
Nutritional treatment during 24 h ICU admission, *n* (%)			
AGI with 2 to 4 stages	283 (25.8)	29 (9.3)	0.348
Nutritional therapy initiation	632 (58)	90 (62)	0.321
Nutritional routine			0.015
EN	454 (71)	34 (57)	
PN	172 (27)	26 (43)	
Oral	16 (2)	0 (0)	
The way of EN delivery			0.027
Gastric feeding	442 (97)	30 (88)	
Postpyloric feeding	9 (2)	3 (9)	
PEG/J or jejunostomy feeding	5 (1)	1 (3)	
EN delivery infusion way			0.176
Continuous	426 (95)	34 (100)	
Discontinuous	23 (5)	0 (0)	
The adverse aspect of feeding during 24 h ICU admission, *n* (%)			
Nausea	200 (18)	30 (21)	0.657
Vomiting	69 (6)	13 (9)	0.303
Aspiration	173 (16)	25 (17)	0.722
Abdominal pain	78 (7)	11 (8)	0.654
Bloating	41 (4)	6 (4)	0.267
Tolerability assessment	574 (52)	68 (47)	0.273
Gastric residue, Median (IQR)	80 (0, 300)	280 (0, 300)	0.073
Feeding interrupt, *n* (%)	188 (17)	35 (24)	0.048

IQR, interquartile range; ICU, Intensive Care Unit; SOFA, Sequential Organ Failure Assessment; APACHE II, Acute Physiology and Chronic Health Evaluation II; GCS, Glasgow Coma Score; WBC, white blood cell; CRP, C-reactive protein; ALB, albumin; AGI, acute gastrointestinal injury; EN, enteral nutrition; PN, parenteral nutrition; PEG/J, percutaneous endoscopic gastrostomy/jejunostomy.

Meanwhile, the features of nutrition treatment were also shown. Compared to the survivors’ group, there was a lower ratio of EN delivery (57% vs. 71%; *p* = 0.015), a higher ratio of post-pyloric feeding (9% vs. 2%; *p* = 0.027), and a higher frequency of feeding interrupt (24% vs. 17%; *p* = 0.048) in the non-survivors group.

### 3.4 Nutrition treatment factors associated with long-term mortality in elderly critical patients

Univariate logistics regression analysis was used to investigate the factors associated with 28-day mortality in critically ill elderly patients. As shown in Table 3, Several factors were significantly associated with long-term mortality. These include age with an OR of 1.047 (95% CI, 1.020–1.075), weight with an OR of 0.975 (95% CI, 0.956–0.995), previous gastrointestinal tumor with an OR of 0.412 (95% CI, 0.237–0.716), APACHE II score with an OR of 1.0385 (95% CI, 1.008–1.070), respiratory rate with an OR of 1.036 (95% CI, 1.007–1.067), lactate with an OR of 1.060 (95% CI, 1.010–1.114), and post-pyloric feeding, 24 h after ICU admission, with an OR of 4.911 (95% CI, 1.263–19.096). Also, when using gastric feeding as a reference, and PN delivery 24 h after ICU admission with OR 2.018 (95% CI 1.176–3.463) and EN delivery as reference.

**Table 3:** Univariate logistics regression analysis for the factors that influenced mortality in elderly critically ill patients.

Parameters	OR	95%CI	*p*
Gender	0.905	0.583–1.406	0.657
Age, years	1.047	1.020–1.075	0.001
Height, cm	1.007	0.980–1.034	0.629
Weight, kg	0.975	0.956–0.995	0.014
ICU diagnosis			
Sepsis	1.022	0.481–2.170	0.955
Septic shock	1.064	0.568–1.995	0.847
Cardiac arrest	4.400	0.601–32.200	0.145
Severe pancreatitis	3.945	0.538–28.936	0.177
Cerebral disease	1.702	0.992–2.919	0.054
Underlying diseases			
Hypertension	0.971	0.612–1.543	0.902
Diabetes	0.768	0.449–1.315	0.336
Chronic renal disease	1.301	0.696–2.431	0.410
Gastroenteric tumor	0.412	0.237–0.716	0.002
SOFA	1.037	0.975–1.102	0.249
APACHE II	1.038	1.008–1.070	0.013
Temperature, °C	0.968	0.772–1.213	0.775
Mean arterial pressure, mmHg	0.997	0.987–1.007	0.563
Heart rate, beats/min	1.005	0.996–1.014	0.272
Respiratory rate, beats/min	1.036	1.007–1.067	0.015
Platelet, × 10^9^/L	0.999	0.997–1.001	0.347
Total bilirubin, mmol/L	1.001	0.999–1.003	0.562
Creatinine, umol/L	1.000	0.998–1.001	0.575
WBC, × 10^9^/L	1.009	0.978–1.041	0.571
Lymphocyte, %	0.981	0.957–1.006	0.129
CRP, mmol/L	1.002	0.999–1.005	0.129
ALB, mg/dl	0.986	0.955–1.018	0.387
Lactate, mmol/L	1.060	1.010–1.114	0.019
Glucose_min, mmol/L	0.987	0.905–1.077	0.774
Glucose_max, mmol/L	1.025	0.984–1.067	0.244
AGI with 1 stage as reference			0.480
AGI with 2 stage	1.243	0.744–2.077	0.406
AGI with 3 stage	1.633	0.714–3.737	0.245
AGI with 4 stage	0.467	0.062–3.493	0.458
Nutritional routine			
EN			0.039
PN	2.018	1.176–3.463	0.011
Oral	0.000	0.000	0.999
The way of EN delivery			
Gastric feeding			0.049
Postpyloric feeding	4.911	1.263–19.096	0.022
PEG/J or jejunostomy feeding	2.947	0.334–26.032	0.331
Abdominal pain	1.514	0.693–3.308	0.298
Bloating	1.677	0.737–3.813	0.218
Gastric residue	1.138	0.554–2.335	0.725
Feeding interrupt	1.080	0.246–4.737	0.919

ICU, Intensive Care Unit; SOFA, Sequential Organ Failure Assessment; APACHE II, acute physiology and chronic health evaluation II; WBC, white blood cell; CRP, C-reactive protein; ALB, albumin; AGI, acute gastrointestinal injury; EN, enteral nutrition; PN, parenteral nutrition; PEG/J, percutaneous endoscopic gastrostomy/jejunostomy.

**Figure 2:**
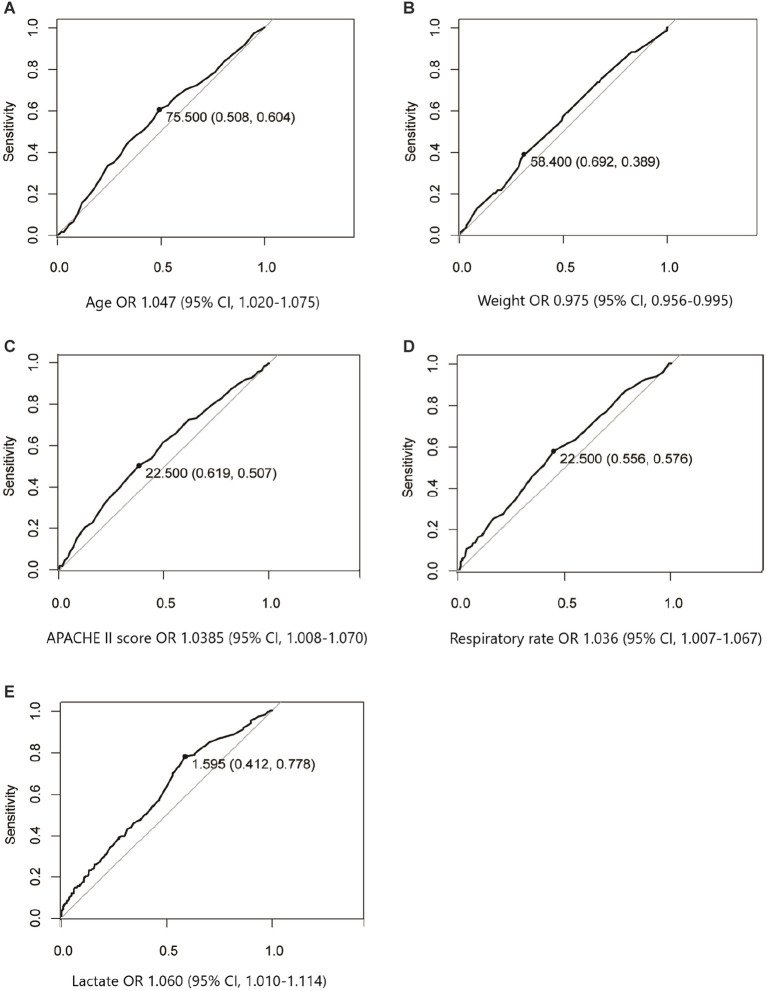
ROC curves were performed to explore the diagnostic efficiency in continuous variables that significantly influenced mortality after univariate logistics regression analysis. The optimal cutoff value associated with survival of age median **(A)**, weight median **(B)**, APACHE II score median **(C)**, respiratory rate median **(D)**, and lactate median **(E)** were 75.5 years old, 58.4 kg, 22.5, 22.5 beats/min, and 1.595 mmoL/L, respectively.

The ROC curves were performed to explore the diagnostic efficiency in continuous variables with a significant difference after univariate logistics regression analysis. As Figure 2 shows, the optimal cutoff value of age median, weight median, APACHE II score median, respiratory rate median, and lactate median was 75.5 years old, 58.4 kg, 22.5, 22.5 beats/min, and 1.595 mmoL/L, respectively.

We then changed the continuous variables with significant differences into dichotomous variables according to the optimal cutoff value. To investigate the independent factors that influenced long-term mortality, all the dichotomous variables with significant differences after univariate logistics regression analysis were entered into the multivariable logistics regression model. Table 4 shows that several factors were significantly independently associated with long-term mortality. These include age above 76 years old with an OR of 2.576 (95% CI, 1.127–5.889), respiratory rate > 22 beats/min, 24 h after ICU admission with an OR of 2.385 (95% CI, 1.101–5.168), lactation >1.5 mmol/L, 24 h after ICU admission with an OR of 7.004 (95% CI, 2.395–20.717). In addition, PN delivery is 24 h after ICU admission with an OR of 5.401 (95% CI, 1.175–24.821) when using EN delivery as reference.

**Table 4:** Multi-logistics regression analysis for the factors that influenced mortality in elderly critically ill patients.

Parameters	OR	95%CI	*p*
Age > 76 years	2.576	1.127–5.889	0.025
Respiratory rate > 22 beats/min at 24 h ICU admission	2.385	1.101–5.168	0.028
Lactate >1.5 mmol/L at 24 h ICU admission	7.044	2.395–20.717	<0.001
EN delivery at 24 h ICU admission			0.038
PN delivery at 24 h ICU admission	5.401	1.175–24.821	0.030
Oral delivery	5.651	0.570–56.018	0.139

ICU, Intensive Care Unit; EN, enteral nutrition; PN, parenteral nutrition.

## 4 Discussion

The multi-center prospective study, which involved 128 ICUs from different hospitals in mainland China, was the first to determine the characteristics of nutrition treatment in elderly critical patients. We included 1,238 elderly critical patients with a median age of 76 years and further investigated the effect of nutrition patterns and nutrition programs on 28-day all-cause mortality in elderly patients who are critically ill. Our data suggested that delivery and therapy of nutrition was an independent risk factor for a 28-day survival and that enteral nutrition could improve the 28-day mortality.

Several previous studies on nutritional therapy in critically ill patients have not reached consistent conclusions due to the heterogeneity of patient’s conditions and the complexity of clinical diagnosis and treatment. In a global study involving 880 units in 46 countries, only 10% of patients received enteral feeding on the first day (13). In our study, 24 h after ICU admission, 58% of elderly critical patients had initiated nutrition therapy, including 34.2% with an EN delivery of nutrition. EN should be started within 24 h in critically ill patients who can sustain voluntary ingestion according to clinical practice guidelines (14). Furthermore, we made a personalized nutrition therapy according to the assessment of the nutrition tolerance of each patient according to the AGI scale. Additionally, EN delivery was also involved in our study. In this study, 96% of patients with EN had gastric feeding and 7% experienced AGI stages 3 to 4. We found that a lower rate of EN (57% vs. 71%), higher rates of post-pyloric feeding (9% vs. 2%), and feeding interrupt (9% vs. 2%) were associated with an increased risk of 28-day mortality. When using EN delivery as a reference, 24 h after ICU admission in multivariable analysis, PN delivery, 24 h after ICU admission, was independently associated with long-term mortality. A cohort study involving 3,500 patients showed that within 2 years, enteral nutrition had a better prognosis than PN in patients with and without malignant diseases (15). Multivariable logistics regression analysis showed that age > 76 years during 24 h of ICU admission, respiratory rate > 22 beats/min, lactate >1.5 mmol/L, and early PN feeding delivery at 24 h after ICU admission had independently increased the risk of mortality and were the independent risk factors for mortality in elderly patients who are critically ill when using EN as a reference.

In a worldwide study involving 9,777 critical adult patients from 46 countries and 880 units, oral feeding was very common; 50% of patients had enteral feeding on the first day, which increased to 75% of patients after 5 days, and parenteral nutrition was administered to approximately 10% of patients (13). This indicates that enteral nutrition that leads to AGI in the early stage is relatively common. The conclusion of this study is similar to the current study. In another multinational study conducted in Latin America, EN nutrition therapy occurred in 79.9% of critically ill patients, PN alone (9.4%), and EN + PN (10.7%). Meanwhile, 59.7% of patients received >90% of the estimated daily target within 24 h after ICU admission (16). The conclusion of this study is also similar to the current study. Due to the poor immune function in the elderly, the body condition is complex and diverse, and the nutritional status and gastrointestinal function appear to be impaired, contributing to malnutrition and frailty with poor recovery and even disastrous prognosis in elderly patients who are critically ill (17).

Old age and nutritional status are key factors associated with adverse clinical outcomes (18). Therefore, it is important to clarify the status of nutrition treatment in elderly patients who are critically ill. ESPEN guideline detailed that critically ill elderly patients should initiate early EN (within 48 h) rather than delaying it (19). In our multicenter study, 34.2% of elderly patients received EN on the first day, and this percentage increased to 58.3% within 3 days. Parenteral nutrition was prescribed to 16.0% of the patients and decreased to 10.7% within 3 days. The ratio of enteral nutrition was lower than previous studies focused on critically ill elderly patients (13, 20). ESPEN guidelines on clinical nutrition in the intensive care unit recommend an oral diet over EN or PN in critically ill patients who can eat (19). However, in our study, the percentage of oral treatment in elderly patients who are critically ill is very low, which indicates that most elderly patients who are critically ill are unable to provide their nutrition. At the same time, the ratio of PN is higher than that of critically ill adult patients. Approximately 4.16% of the patients in our study had intestinal functional intolerance and required post-pyloric feeding, and 25.2% of patients had AGI at 2 to 4 stages and decreased to 5.1% after 3 days. The rates of feeding complications, including aspiration, were high in elderly patients who were critically ill 24 h after ICU admission, and the symptoms of abdominal problems were high 48 h after ICU admission.

A meta-analysis, including 18 randomized controlled trials focused on the impact of early EN vs. early PN on clinical outcomes in critically ill patients, has concluded that the uses of EN as compared to PN leads to a reduction of infectious complications and shorter ICU stay, but no difference in mortality (21). However, in the NUTRIREA-2 study, compared to early isocaloric PN, early isocaloric EN does not affect the mortality rate or the risk of secondary infections, in adverse, with a greater risk of digestive complications in critically ill adults with shock (22). Therefore, different nutritional support strategies are needed for different critically ill patients (7, 17, 23–25). Nutritional screening in the intensive care unit (ICU) requires an understanding of two key points, the nutritional status and severity of the disease at admission, as well as different treatment measures and the duration of organ support (26). ESPEN guideline for clinical nutrition and hydration in geriatrics has indicated that even short-term starvation in the acutely ill older person leads to loss of lean body mass, which can be critical, especially in older patients, so it has suggested that regardless of the nutritional status and severity of the disease upon admission, if possibly, PN should be initiated immediately in older patients (8). In our study, for those critically ill elderly patients, after multivariable logistics regression analysis, we found that when EN delivery was used as a reference, PN delivery 24 h after ICU admission was the independent risk factor associated with long-term mortality with OR 5.401 (95% CI, 1.175–24.821). The main reasons for the worse impact of early PN on clinical outcomes are that it may bring caloric overfeeding and risk of ICU infection.

A randomized controlled trial by White et al. has compared early post-pyloric versus early gastric feeding in ventilated intensive care patients, and the result showed that early post-pyloric feeding had no advantage over early gastric feeding (27). The results indicated that feeding mode did not affect prognosis but rather complications. Zhu et al. conducted a single-center randomized trial to explore gastric versus post-pyloric feeding in elderly patients (age ≥ 75 years) on mechanical ventilation and found that compared to gastric EN, post-pyloric EN reduced the risk of ventilator-associated pneumonia (VAP), but did not affect mortality (28). Therefore, gastric feeding is recommended as an initial EN delivery in critically ill elderly patients with high-risk factors of aspiration (7, 19, 29, 30). In our multi-center prospective study, we found that the incidence of early post-pyloric feeding in elderly patients who are critically ill is rare regardless of being in the survivor or non-survivor groups (2% vs. 9%). However, the reasons might be that the clinical physician might strictly evaluate EN tolerance in elderly patients who are critically ill while adhering to EN protocols based on current nutrition guidelines and then those with a high risk of aspiration.

Our study has some limitations. First, regardless of the multi-center prospective study with 1,238 elderly patients, this study, in one part, reflects the nutritional status of elderly patients who are critically ill from different ICU units in China. In the other part, the heterogeneity of the study derived from the treatment practices in the population included is very different in different units. So, to identify the effect of nutrition treatment on mortality, we used multivariable logistics regression analysis to control the confounding bias. Second, this study only shows some associations between nutrition treatment and mortality. Therefore, further study needs to delve into the influencing factors of nutritional support and the causes of nutritional intolerance in elderly patients who are critically ill. Our study mainly targeted elderly patients. Age is an important factor in increasing ICU mortality, and nutrition is also one of the most important links. Last but not least, nutritional status at admission is very important, but BMI was not collected in our study design. Therefore, nutritional status at admission may be a potential confounding factor for a 28-day survival prognosis.

## 5 Conclusion

This multi-center prospective study describes clinical characteristics, the mode and timing of nutrition treatment, frequency of AGI, and adverse effects of nutrition in elderly ICU patients. According to this survey, ICU patients with early PN delivery, older age, faster respiratory rate, and higher lactate level may experience poor prognosis.

## Clinical relevancy statement

The status of nutrition treatment and feeding-influenced mortality remains uncertain in elderly patients in the intensive care unit (ICU). Our article describes that in elderly critically ill patients, the proportion of enteral nutrition (EN) delivery at an early stage is low and the choice of early Parenteral nutrition (PN) feeding seems to be associated with disastrous clinical outcomes.

## Data availability statement

The raw data supporting the conclusions of this article will be made available by the authors, without undue reservation.

## Ethics statement

This study was approved by the medical ethics committee of Nanjing General Hospital of Nanjing Military Command (2017NZKY-010-01). The studies were conducted in accordance with the local legislation and institutional requirements. The participants provided their written informed consent to participate in this study.

## Author contributions

WC: Conceptualization, Writing – original draft. MP: Data curation, Methodology, Writing – review & editing. ZY: Data curation, Formal analysis, Writing – review & editing. YA: Data curation, Formal analysis. ZL: Funding acquisition, Software, Supervision, Validation, Visualization, Writing – review & editing.

## Supplementary material

SUPPLEMENTARY FIGURE S1 Flowchart of patient selection.
