# Supplementary figures and images for: The mode and timing of administrating nutritional treatment of critically ill elderly patients in intensive care units: a multicenter prospective study
Source: Front Med (Lausanne). 2024 Feb 7;11:1321599. doi: 10.3389/fmed.2024.1321599 (PMC10879295; doi:10.3389/fmed.2024.1321599)

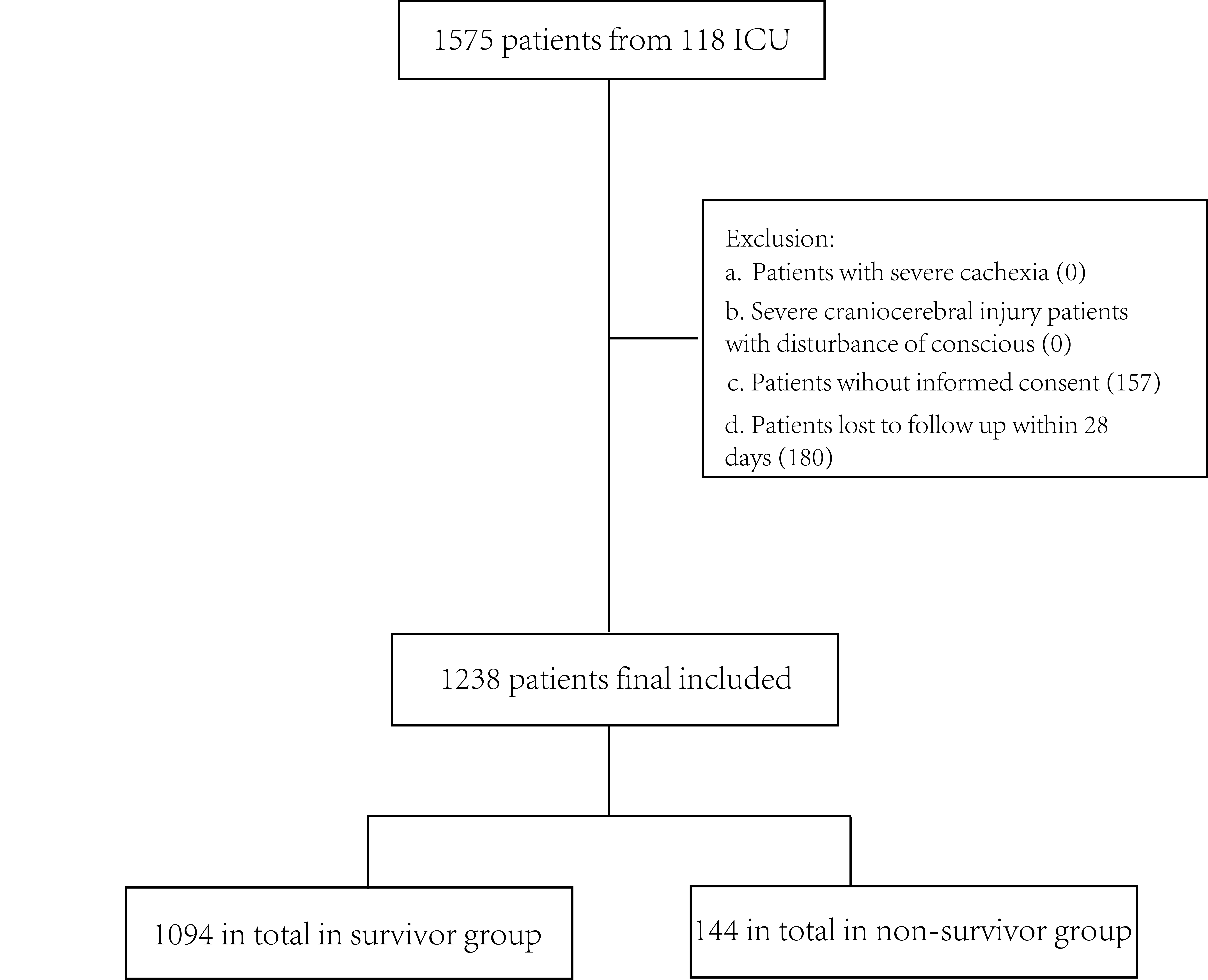

Supplement: SUPPLEMENTARY FIGURE S1 — Flowchart of patient selection. [file Image_1.TIF]
